# Epigenetic based synthetic lethal strategies in human cancers

**DOI:** 10.1186/s40364-020-00224-1

**Published:** 2020-09-15

**Authors:** Aiai Gao, Mingzhou Guo

**Affiliations:** 1 Department of Gastroenterology and Hepatology, Chinese PLA General Hospital, #28 Fuxing Road, Beijing, 100853 China; 2 Henan Key Laboratory for Esophageal Cancer Research, Zhengzhou University, 40 Daxue Road, Zhengzhou, 450052 Henan China; 3 State Key Laboratory of Kidney Diseases, Chinese PLA General Hospital, #28 Fuxing Road, Beijing, 100853 China

**Keywords:** DNA damage repair, Synthetic lethality, Epigenetics, BRCA1/2, PARP inhibitor

## Abstract

Over the past decades, it is recognized that loss of DNA damage repair (DDR) pathways is an early and frequent event in tumorigenesis, occurring in 40-50% of many cancer types. The basis of synthetic lethality in cancer therapy is DDR deficient cancers dependent on backup DNA repair pathways. In cancer, the concept of synthetic lethality has been extended to pairs of genes, in which inactivation of one by deletion or mutation and pharmacological inhibition of the other leads to death of cancer cells whereas normal cells are spared the effect of the drug. The paradigm study is to induce cell death by inhibiting PARP in BRCA1/2 defective cells. Since the successful application of PARP inhibitor, a growing number of developed DDR inhibitors are ongoing in preclinical and clinical testing, including ATM, ATR, CHK1/2 and WEE1 inhibitors. Combination of PARP inhibitors and other DDR inhibitors, or combination of multiple components of the same pathway may have great potential synthetic lethality efficiency. As epigenetics joins Knudson’s two hit theory, silencing of DDR genes by aberrant epigenetic changes provide new opportunities for synthetic lethal therapy in cancer. Understanding the causative epigenetic changes of loss-of-function has led to the development of novel therapeutic agents in cancer. DDR and related genes were found frequently methylated in human cancers, including BRCA1/2, MGMT, WRN, MLH1, CHFR, P16 and APC. Both genetic and epigenetic alterations may serve as synthetic lethal therapeutic markers.

## Introduction

The integrity of DNA is continually challenged by a variety of agents and processes that either alter the DNA sequence directly or cause mutation when DNA is suboptimally repaired. The ultraviolet component of sunlight can cause up to 1X10^5^ DNA lesions per cell per day [1]. For example, ionizing radiation can cause single-strand breaks (SSBs) and double-strand breaks (DSBs). If misrepaired, these breaks can induce mutations and lead to widespread structural rearrangement of the genome. DNA damage and mutation may be induced by environmental factors, including cigarette smoking, industrial chemicals, mustard gases and chemical therapeutic agents (such as cisplatin, mitomycin C). Reactive oxygen species (ROS) and other metabolites produced by endogenous processes can also induce DNA damaging [2]. According to the type of DNA lesion they process, DNA damage repair/response (DDR) may be divided into different pathways, which are functionally interwoven. Most of the subtle changes to DNA, such as oxidative lesions, alkylation products and SSBs, are repaired by base excision repair (BER) [3]. Whereas some of the bulkier single strand lesions that distort the DNA helical structure, such as those caused by ultraviolet light, are processed by nucleotide excision repair (NER) [4]. The major DDR mechanisms that cope with DSBs are homologous recombination repair (HR) and non-homologous end joining repair (NHEJ) [5]. HR is an error-free repair and mainly acts in the S and G2 phase of cell cycle. The major components involved in HR include BRCA1, BRCA2, RAD51 and PALB2 genes. NHEJ occurs throughout the cell cycle. NHEJ mediates repair by directly ligating the end of a DSB together and may cause DNA deletion or mutation at the DSB site. Mismatch repair (MMR) deals primarily with dNTP misincorporation and formation of ‘insertion and deletion’ loops that form during DNA replication. Key proteins involved in this process including MLH1 and MSH2 [6, 7].

Defects in DDR can lead to an increase in genomic instability, which is one of carcinogenesis mechanisms in various cancers. However, DDR defects can be exploited in cancer therapy because excessive genomic instability itself can have lethal consequences by inducing deadly mutations, mitotic catastrophe, or chromothripsis [8]. Among the variety of types of DNA damage, the most deleterious is the DNA DSB. Cancer chemotherapeutic agents and radiotherapy exert their cytotoxic effects by inducing DNA DSBs [7]. DDR components are often defective in cancer, but the DDR comprises interacting/crosstalking pathways, and defects in one can be compensated by alternative pathways. Such compensatory pathways obstruct effective cancer treatment [9]. For example, DSBs are predominantly repaired by the NHEJ pathway in G1 phase of cell cycle and by HR in S-G2 phases. Microhomology mediated end joining (MMEJ) is a “backup” DSB repair pathway in the event that NHEJ or HR are compromised. HR and MMEJ share the same substrate, a resected DSB that contains a 3′ single-stranded DNA (ssDNA) overhang bound by replication protein A (RPA). Thus, if HR is defect, MMEJ is the favored option to repair resected DSBs [10]. The concept of synthetic lethality was first described in fruit flies, when two single genetic defects, loss-of-function events, either alone had no effect on viability, but when combined resulted in lethality [11]. Over the past decades, it is recognized that loss of DNA repair pathways is an early and frequent event in tumorigenesis, occurring in 40-50% of many cancer types [10]. However, DDR deficient cancers become critically dependent on backup DNA repair pathways, which present an “Achilles heel” that can be targeted to eliminate cancer cells. This is the basis of synthetic lethality. In 2005, two groups discovered a synthetic lethal interaction between PARP inhibition and mutations in BRCA1 or BRCA2 [12, 13]. The concept of “BRCAness” was originally meant that the phenotypes of some sporadic tumors share with “familial BRCA cancers”. It was broadened by finding more functional “BRCAness” biomarkers [14]. In cancer, the concept of synthetic lethality has been extended to pairs of genes, in which inactivation of one by deletion or mutation and pharmacological inhibition of the other leads to death of cancer cells whereas normal cells are spared the effect of the drug [15]. With great understanding of the biology of DDR, more small molecules are being developed as new anticancer therapies by targeting DDR. There are approximately 450 genes coding for proteins involved in the DDR [16]. Many cancer type specific and pan-cancer targets were discovered through Proof-of-concept classical synthetic lethal screening by small interfering RNA (siRNA) and CRISPR technology [15]. In addition, epigenetics plays an important role in silencing tumor suppressor gene expression, including DDR genes. Thus, numerous potential targets need to be identified in various cancers. This review mainly focused on applying “synthetic lethal” to human cancers with “BRCAness” and beyond caused by loss-of-function with defect of genetics or epigenetics.

## DNA damage checkpoints and DDR inhibitors

DDR is composed of sensor proteins that detect and signal DNA damage to downstream effectors that, in turn, arrest cell cycle progression and promote repair. In response to DNA damage, cell cycle checkpoints can be activated in G1 phase, in S phase and at the G2/M transition [17–19]. Ataxia Telangiectasia Mutated (ATM) kinase is activated by DNA DSB and triggers the G1 checkpoint by phosphorylating and activating the checkpoint kinase 2 (Chk2) [20]. Chk2 inhibits Cdc25A preventing cells from proceeding into S phase [21]. Of note, the G1 checkpoint is critically dependent on p53. The loss of G1 checkpoint control is almost ubiquitous in cancer, making cancer cells more reliant on the S and G2/M checkpoints. When DNA damage occurs in S phase, the intra S phase checkpoint is activated to prevent further replication [22]. Ataxia Telangiectasia and Rad3 related (ATR) kinase is activated by DNA damage, through activating checkpoint kinase 1 (Chk1), and then induces Cdc25A proteosomal degradation to block further progression through S phase [23]. ATR and Chk1 also trigger the G2/M checkpoint, which prevents cells with damaged DNA from entering mitosis [24]. ATR inhibits cyclin B/Cdk1 activation by stimulating the Wee1 (a Cdk1 inhibitory kinase) and inhibiting Cdc25c via Chk1 [17]. When cells with irreparable DNA damage are forced to enter into mitosis, they undergo permanent growth arrest or cell death through a so–called mitotic castrophe mechanism [25]. Forced entry of DNA-damaged cells into mitosis may provide a substantial increase in therapeutic efficacy.

A well-recognized sensor of DNA damage is the protein PARP, which is best known for its role in BER and repair of DNA SSBs. All PARP inhibitors (PARPi) interact with the binding site of the PARP enzyme cofactor, β-nicotinamide adenine dinucleotide (β-NAD+), in the catalytic domain of PARP1 and PARP2, including olaparib and niraparib [26]. Both BRCA1 and BRCA2 proteins are critical to the repair of DSBs by HR. Based on the synthetic lethal interaction between PARP inhibition and BRCA1 or BRCA2 mutation, Farmer and Bryant developed a novel treatment strategy for BRCA-mutant tumors [12, 13]. Applying the concept of synthetic lethality, preclinical PARPi studies demonstrated that PARPi were able to selectively target HR deficient cells [27]. Data from phase II trials of women with BRCAmut ovarian cancer using olaparib at a dose of 400 mg orally twice daily demonstrated RECIST (Response Evaluation Criteria in Solid Tumors) response rates of 30-41% [27, 28]. The phase II trial by Kaufman et al. demonstrated a 31% response rate and an additional 49% stable disease rate in their study subset of 193 women with platinum-resistant ovarian cancer [29]. A phase II study of olaparib in patients with metastatic, hormone resistant prostate cancer showed promising results. Patients whose tumors harboring homologous deletions, mutations or both in DDR genes had a response rate of 33% [30]. More specific and active PARPi are in developing and ongoing studies performing in breast, pancreatic and other cancers.

NER is a multistep DNA repair mechanism involving more than 30 different proteins to excise approximately 30 bases on a damaged DNA strand and to synthesize new DNA. One of the approaches aims at blocking the interaction between the different elements of the NER pathway, thus preventing the repair from being completed. The interaction of XPA with ERCC1 proved to be viable as druggable targets for cancer treatments. F06 inhibits NER by targeting XPF to reduce the interaction between ERCC1 and XPF [31]. ET-743, another NER inhibitor, was recently approved for the treatment of soft tissue sarcomas, ovarian cancer and is currently in clinical trials for the treatment of breast, prostate, and pediatric sarcomas [32]. Although NHEJ is considered to be the main pathway for repair of IR-induced DSBs, relatively little success has been observed with inhibitors targeting the main proteins in this pathway. Three DNA-PK related inhibitors MSC2490484A, CC-122, and CC-115 (DNA-PK and MTOR dual inhibitor) are currently being investigated in phase I clinical trial either for solid tumors, non-Hodgkin lymphoma, multiple myeloma or hematologic malignancies [33]. And a phase II clinical trial of CC-115 is onging in glioblastoma (NCT02977780). ATM is activated following DSBs and plays a major role in the DDR to DSBs caused by IR. ATM inhibitors were developed by different groups, including KU-55933, KU-60019 and KU-59403 [34–36]. ATR is primarily activated by SSBs and responds to DNA replication stress and is therefore active in the S and G2 phases of the cell cycle. Previous studies have demonstrated that alterations in canonical DDR/cell cycle checkpoint genes (ERCC1, XRCC1, CDC25A and ATM) have the potential to act as predictive biomarkers of single-agent ATRi sensitivity [37–39]. In preclinical studies, ATRi (VE-821) enhances the cytotoxic effects of a number of DNA damaging agents in tumor cells that have defects in the ATM/p53 pathway, including cisplatin, topotecan, and veliparib [40]. Although > 1000 compounds have been evaluated as potential ATR inhibitors, only a few have exhibited “drug-like” properties. VX-970, VX-803, BAY1895344 and AZD6738 are currently in clinical studies [41]. Chk1 and Chk2 activation occurs through distinct mechanisms. Chk1 activation is primarily downstream of ATR in response to genotoxic insults. Chk2 is activated primarily by ATM in response to DSBs [42]. Chk1 is the primary effector of the intra-S and G2/M phase checkpoints, whereas Chk2 plays an accessory role, exerting a partial influence on the intra-S and G1/S checkpoints [43]. Chk1 exerts its function often through interacting with other proteins. Numerous proteins have been reported to interact with Chk1 [44]. In addition, crosstalk takes place between the ATR-Chk1 and ATM-Chk2 signaling cascades. Chk1 was initially thought to function as a tumor suppressor, and numerous efforts were made to look for Chk1 mutations in human tumors. However, so far no homozygous loss-of-function mutation of Chk1 has been detected in a wide range of human tumors [45, 46]. Chk1 inhibitors have been in development for two decades [47]. Chk1 inhibitor monotherapy often demonstrates limited efficacy and in general, must be combined with other agents. Evidence from the published clinical trials suggests that some Chk1 inhibitors can be administered safely, but when they are combined with traditional cytotoxic DNA damaging agents, the normal tissue toxicities outweigh the very modest gains in therapeutic efficacy. A variety of Chk1 and/or chk2 inhibitors are under active preclinical development, including EXEL-9844, CEP-3891, PD-321852, Chir-124, CCT241533, LY2606368 [48]. The combination of Chk1 inhibitors with other signaling regulators may be a better therapeutic strategy [49, 50]. In humans, the WEE kinase family consists of three kinases, including PKMYT1 (membrane-associated tyrosine- and threonine-specific cdc2-inhibitory kinase), WEE1 and WEE1B (WEE2) [51]. WEE1B expresses during early embryogenesis, and the expression is significantly reduced after fertilization [52]. PKMYT1 and WEE1 negatively regulate the cell cycle via the phosphorylation of CDK1. Both kinases are considered as main gatekeepers of the G2 cell-cycle checkpoint. Due to mutations in the p53 network, many cancer cells have defective G1 checkpoint mechanisms, which can result in increased DNA damage at the G2 checkpoint compared to normal cells. Cells with intact G1 checkpoint arrest, such as normal cells or cancer cells with intact p53 signaling, are less dependent on the G2 checkpoint arrest. Inhibition of PKMYT1 and WEE1 is particularly effective in cells with deficient p53 signaling [53, 54]. MK-1775 is the most potent and highly selective inhibitor of WEE1 and has recently reached phase I clinical trials [51]. Overexpression of POLθwas found in multiple HR-proficient tumor types such as lung, gastric and colorectal cancer, and was associated with adverse clinical outcomes [55]. The reason why POLθis upregulated and associated with poor outcomes in many tumors is not well understood. The possibility is that POLθrepairs spontaneous DNA damage present in cancer cells and therefore affords them a growth advantage [56]. POLθinhibitors could synergize with PARPi for the treatment of HR-deficient cancers [10].

## Genetic based synthetic lethal therapy in cancer

Currently, most of targeting therapies in cancer are directly targeted at activated oncogenes or “gain of function” genetic aberrations, including gene mutation, amplification and fusion. Even though some of these strategies are very successful, such as EGFR and ALK inhibitors in lung cancer therapy, acquired resistance remains a major barrier to treatment [57]. Unfortunately, not all identified mutations or aberrant expressions can be directly targeted. Such as ‘loss-of-function’ or loss of expression by inactivating gene mutations are hard to restore activities pharmacologically, and less success has yet been achieved [58]. Notably, synthetic lethality strategy allows the therapeutic exploitation of non-druggable mutated tumor suppressor genes and directly difficult-to-target (hard-druggable) oncogenes, via targeting their synthetic lethality partners [59]. Synthetic lethality approaches can achieve the effect of “target damage” by taking advantage of inherent differences between cancer cells and normal cells, which is not feasible with traditional chemotherapy [60]. Given that differences of genomic features between cancer cells and healthy cells, Hartwell was the first to use traditional genetic approaches, genomic information and model organisms for identifying and validating new targets for drugs that would selectively kill tumor cells with a particular molecular [26]. Subsequently, siRNA and CRISPR screenings have been developed to detect synthetic lethal gene pairs in human cells [15]. In recent years, Jerby-Arnon et al. proposed a computational and bioinformatics approach, named data mining synthetic lethality identification pipeline (DAISY), to identify genome-wide synthetic lethal interactions, by analyzing large volumes of cancer genomic data. They applied DAISY to identify all gene pairs that are likely to be synthetic lethal in cancer, resulting in a synthetic lethal network of 2077 genes and 2816 synthetic lethal interactions, and an synthetic dosage lethal (SDL) network of 3158 genes and 3635 SDL interactions [61]. With the help of these advanced screening tools, more novel candidate targets are expected to be discovered. The best-characterized synthetic lethality relationship is between BRCA1 or BRCA2 mutation and PARP inhibition. Several synthetic lethal combinations have been discovered, including Wee1 inhibitor (WEE1i) in p53-defecient cells, ATM inhibitor AZD0156 in conjunction with olaparib or irinotecan (a topoisomerase inhibitor) [62, 63]. It is well established that the genomes of all cancers carry somatic mutations, and advances in DNA sequencing technologies have made it possible to identify thousands of individual somatic mutations in a single cancer genome [64]. The mutation rate is not constant throughout the genome but differs~ 5-fold, and a higher mutation rate is typically seen in late replicating and low transcribing genes [65]. Notably, 20% of tumors from a pan-cancer analysis identified subclonal mutations in the BRCA1/2 pathway [66]. A mutational signature associated with defective HR was first identified in BRCA1/2 germline mutant breast cancers [67], and later in ovarian, pancreatic, and gastric cancers [68–71]. As shown in Table 1, mutant DDR, cell cycle control and tumor suppressor genes are including RAD51, PALB2, P53, ATM, MGMT and Others [26]. Genes that result in a synergistic effect are commonly interpreted as working in compensatory pathways. The identification of such functional networks is particularly important for understanding cancer-related signaling pathways because the heterogeneity in the genetic background of cancers is often associated with the connected pathways that might provide multiple potential rewiring mechanisms.

**Table 1:** Genetic defects of DDR in cancer and DDR inhibitors

pathway	gene	genetic alterations	drugs	Refs
BER	APE1	SNP		[72]
	OGG1	missense, frameshift, deletion, SNP		[73]
	Pol β	frameshift, Splicing, missense		[74]
	XRCC1	nonsense, missense, SNP	PARP, ATM, DNA-PKcs inhibitors	[75, 76]
	Neil1	deletion, point mutation, SNP		[77]
NER	XPC	SNP		[78]
	DDB1	missense, splicing, deletion		[79]
	ERCC1	SNP	ATR, CHEK1 inhibitors	[37, 78]
	ERCC2(XPD)	missense, SNP, homozygous deletion		[80, 81]
	ERCC4(XPF)	missense		[79]
	ERCC5(XPG)	missense		[79]
	ERCC6	missense, nonsense, splicing		[79]
	XPA	homozygous deletion, SNP		[79, 82]
MMR	MLH1	deletion, missense, nonsense, splicing	POLG, ATR inhibitor	[83, 84]
	MSH2	deletion, nonsense, rearrangements	POLB inhibitor	[83–85]
	MSH6	point mutation	DHFR, POLB, POLG inhibitor	[85, 86]
	EPCAM	deletion		[85]
	PMS2	point mutation		[85]
HRR	BRCA	truncating, missense, large rearrangements	PARP, APE1, ATM, DNA-PKcs inhibitors	[87–89]
	RAD51(RAD51B, RAD51C, RAD51D)	frameshift indels, splicing, nonsense, missense.	PARP inhibitor	[90]
	PALB2(FANCN)	frameshift, nonsense, splicing, deletion	PARP inhibitor	[91, 92]
	XRCC2	SNP		[93]
	XRCC3	SNP		[93]
	MSH3	In-frame deletion, frameshift, missense	DNA-PKcs inhibitors	[94]
FA pathway	FANC genes	missense, deletion, frameshift, SNP	PARP inhibitor	[95, 96]
NHEJ	XRCC4	SNP		[97]
	LIG4	SNP	PARP inhibitor	[98, 99]
Cycle checkpoints	ATR/Chk1	SNP, insertion, deletion	APE1, Wee1, ATM, Chk1 inhibitor	[43, 88, 100–103]
	ATM/Chk2	SNP, nonsense, splicing, frameshift	DNA-PKcs, PARP, polθ, MEK inhibitors	[43, 103, 104]
Others	CDK12	deletion, missense, frameshift	PARP inhibitor	[105]
	BAP1	truncating, missense	PARP inhibitor	[106]
	P53	nonsense, missense	ATR, Chk inhibitor	[107, 108]
	PTEN	Mutation, deletion	PARP inhibitor	[109]

## Epigenetic silencing of DDR and related genes in human cancers

Accumulation of genetic and epigenetic alterations is regarded as a major factor for cancer initiation and progression, and aberrant epigenetic changes occur more frequently than gene mutations in human cancers [110, 111]. Epigenetic regulation of gene expression depends mainly on DNA methylation, histone modification and noncoding RNA. The regulators of “epigenetic machinery” are divided into “writers” (enzymes that establish DNA methylation or histone modification), “erasers” (proteins that remove these marks) and “readers” (proteins that bind to modifications and facilitate epigenetic effects) [112]. A lot of inhibitors, targeting “epigenetic machinery”, are ongoing clinical trials [111]. Despite their promise, there are many challenges to be resolved for efficient use of epidrugs in the treatment of human cancer, including the lack of specificity of epidrugs, disappointing success in solid tumors and the acquisition of drug chemo-resistance leading to higher risk of tumor relapse. As lack of tumor specific histone modification detection markers, the efficiency of targeting histone modifier therapy remains very limited, even though a number of clinical trials are ongoing [111]. The best studied epigenetic modification is DNA methylation, as the nature of DNA is stable and the reliable detection technologies. Tumor suppressor gene promoter region methylation is frequently found in various human cancers. DNA methylation may serve as early detection, prognostic, chemo-radio-sensitive markers, and therapeutic targets [113]. Noncoding RNAs are functional RNA molecules that do not code for proteins. They are divided into different classes based on size, including siRNAs, miRNA, piRNAs and lncRNA [111]. Each class of RNA performs different endogenous functions, providing a variety of opportunities and challenges for drug discovery. New methods of design for the creation of artificial microRNAs as well as new systems of delivery by nanoparticles have been developed. However, the difficulties of a real tumor-specific delivery still represent an obstacle for the application of these methodologies in cancer therapy [114]. The field of lncRNAs is at its infancy. LncRNAs are poorly conserved across different species, therefore, the structural and functional information as well as the promising therapeutic strategies developed using in vitro and animal models may not be easily extended to human application [115]. The application of noncoding RNA in cancer synthetic lethality is very limited. Srinivasan et al. found that miR223-3p induced synthetic lethality in BRCA1- and BAP1-deficient malignancies by inhibiting alternative NHEJ signaling [116].

Understanding the causative epigenetic changes of loss-of-function has led to the development of novel therapeutic agents in cancer. The expression of DNA damage repair genes was found frequently silenced by promoter region methylation in various cancers. Silencing of DDR and cell cycle related genes provide opportunities for synthetic lethal therapeutic strategies in human cancers [6, 112]. Defects of DDR may cause gene mutation and further induce carcinogenesis. Accumulation of DDR gene hypermethylation was found in the multistep of esophageal carcinogenesis and progression from normal tissue to different types of dysplasia and invasive cancer, including MGMT, MLH1, APC and BRCA1 [110]. Increased methylation of DDR and cell cycle related genes was also found progressively in the different types of pancreatic cancer from noninvasive intraductal papillary mucinous neoplasms (IPMN), IPMN with carcinoma in situ, IPMN with microinvasion and infiltrative IPMN with associated adenocarcinoma, including APC, hMLH1, MGMT, BRCA1, p14 and P16 [117]. These results suggest that synthetic lethality based on DDR methylation may apply to cancer prevention. DDR genes are more commonly found methylated in invasive cancers. Methylation of WRN was found in 37.9% of colorectal cancer (CRC), 37.5% of non-small cell lung cancer (NSCLC), 25% of gastric, 20% of prostate, 17.2% of breast, 12.5% of thyroid cancer, 23.7% of non-Hodgkin lymphoma, 9.5% of acute lymphoblastic leukemia, 4.8% of acute myeloblastic leukemia, 33.3% of chondrosarcomas and 11.1% of osteosarcomas. WRN methylation is a sensitive marker for prediction of irinotecan in colorectal cancer [118]. Methylation of SLFN11 was found in 55.47% of CRC, 39% of papillary serous ovarian cancer, 29.9% of gastric cancer and 13.6% of NSCLCs. Methylation of SLFN11 reduced the sensitivity to cisplatin [119–121]. MLH1 was found methylated in 21.6% of gastric cancer patients and methylation of MLH1 was associated to oxaliplatin resistance [122]. XRCC1 was methylated in 76.4% of gastric cancer [123]. MED1, encoding a base excision repair enzyme, was methylated in 24% of CRC [124]. ERCC1 was methylated in 37.5% of gliomas, XPC was methylated in 34% of lung cancer and 32.45% of bladder cancers, XPG was methylated in 19% of ovarian cancers [125–128]. In NSCLCs, BRCA1, BRCA2, and XRCC5 (ku80 encoding gene) was reported to be methylated in 30, 42 and 20% [129]. Methylation of MGMT is the most studied DDR gene for chemo-sensitivity in various cancers [6, 130, 131]. GPX and GSTPi were also found frequently methylated in various tumors [6, 132].

CHFR, RASSF1A, P14, P15 and P16 genes are directly involved in cell cycle regulation and silenced by frequent methylation in different cancers. Other genes, including DACT2, SOX17, CDX2, NKD2, HIN1, IGFBPL1, TMEM176A, HOXD10, SFRP1, GATA4, GATA5, CDH1, APC, DACH1, were reported frequently methylated and indirectly involved in cell cycle regulation through Wnt, PI3K-AKT, ERK, TGF-beta and other signaling pathways in different cancers [113, 133–140]. Beyond ‘BRCAness’, aberrant epigenetic changes in all these cell cycle regulators may provide new opportunities for synthetic lethal therapy in different cancers. CHFR is involved in G2/M checkpoint regulation, it is frequently methylated in esophageal cancer, gastric cancer and NSCLCs. Methylation of CHFR may serve as a docetaxel-sensitive marker [122, 141, 142]. RASSF1A inhibits G1-S transition and induces G2/M arrest in various cancers. Methylation of RASSF1A was frequently found in esophageal, gastric, lung and other cancers [143]. DACT2 was found frequently methylated in lung, esophageal, breast and other cancers. It may induce G1/S or G2/M checkpoint arrest in different cancers through Wnt signaling [144–147]. NKD2 was methylated in 53.2% of esophageal cancer and silencing of NKD2 activated Wnt signaling to promote G1/S transition [148]. Methylated DDR and related genes in cancers are listed in Table 2.

**Table 2:** Methylation of DDR and related genes in cancers

Pathway	gene	tumor type	methylation frequency	refs
DDR	BRCA1	pancreatic	46%	[149]
		NSCLC	30%	[129]
		ESCC	28%	[110]
		ovarian	16.3%	[150]
	BRCA2	NSCLC	42%	[129]
	MGMT	gliomas	40%	[130]
		NSCLC	30%	[142]
		gastric	9.8%	[122]
	MLH1	CRC	38%	[151]
		ESCC	33%	[110]
		gastric	21.6%	[122]
	MSH2	HPNCC	24%	[152]
		HCC	24.6%	[153]
	WRN	CRC	37.9%	[118]
		NSCLC	37.5%	[118]
		gastric	25%	[118]
		prostate	20%	[118]
	FANCF	cervical	30%	[154]
		ovarian	21%	[154]
		breast	17%	[154]
DDR related	P16	ESCC	52%	[110]
		NSCLC	29%	[142]
	CHFR	ESCC	45%	[141]
		gastric	34.3%	[122]
		NSCLC	10%	[142]
	RASSF1A	gliomas	79.4%	[155]
		cholangiocarcinoma	65%	[156]
		gastric	12.7%	[122]
	SLFN11	CRC	55.47%	[120]
		papillary serous ovarian	39%	[119]
		gastric	29.9%	[121]
		NSCLCs.	13.6%	[119]
	DACT2	ESCC	69%	[145]
		gastric	55.7%	[157]
		breast	49.7%	[146]
		lung	41%	[144]
	NKD2	ESCC	53.2%	[148]
		gastric	53.1%	[158]
		breast	51.4%	[159]
	HIN-1	ESCC	50%	[160]
		NSCLC	48%	[161]
	DACH1	gastric	63.3%	[162]
		ESCC	61.5%	[163]
		HCC	42%	[164]

*NSCLC* Non-small cell lung cancer, *ESCC* Esophageal squamous cell carcinoma, *CRC* Colorectal cancer, *HPNCC* Hereditary non-polyposis, *HCC* Hepatocellular carcinoma

## The interplay of epigenetics and genetics in DNA damage repair genes

Disruption of a key epigenetic regulator by mutation leads to an altered transcriptome, multiplying the effect of the single genetic alteration [165]. DNMT3A is recurrently mutated in acute myeloid leukemia (AML) and other myeloid malignancies [166, 167]. The majority of missense mutations impair the enzymatic activity of TET2, resulting in decreased 5hmC levels and aberrant DNA methylation [168]. Aberrant epigenetic changes may cause genetic abnormality. Epigenetic silencing of DNA repair genes such as MLH1, MGMT, BRCA1, FANCF, CHFR and SLFN11 can lead to gene mutations and genomic instability in cancer cells [112, 120, 169]. Lynch syndrome resulted from germline mutations in mismatch repair genes, primarily MSH2 and MLH1. Approximately 15% of sporadic colorectal cancer patients with microsatellite instability (MSI) were caused by epigenetic silencing of the MLH1 promoter region [151]. Methylation of MGMT in colorectal cancer is associated with G-to-A mutations in the KRAS gene [170]. It is clear that the cancer genome and epigenome influence each other in a multitude of ways. They offer complementary mechanisms to promote oncogenic transformation.

## Epigenetics joins Knudson’s two hit theory

**Fig. 1:**
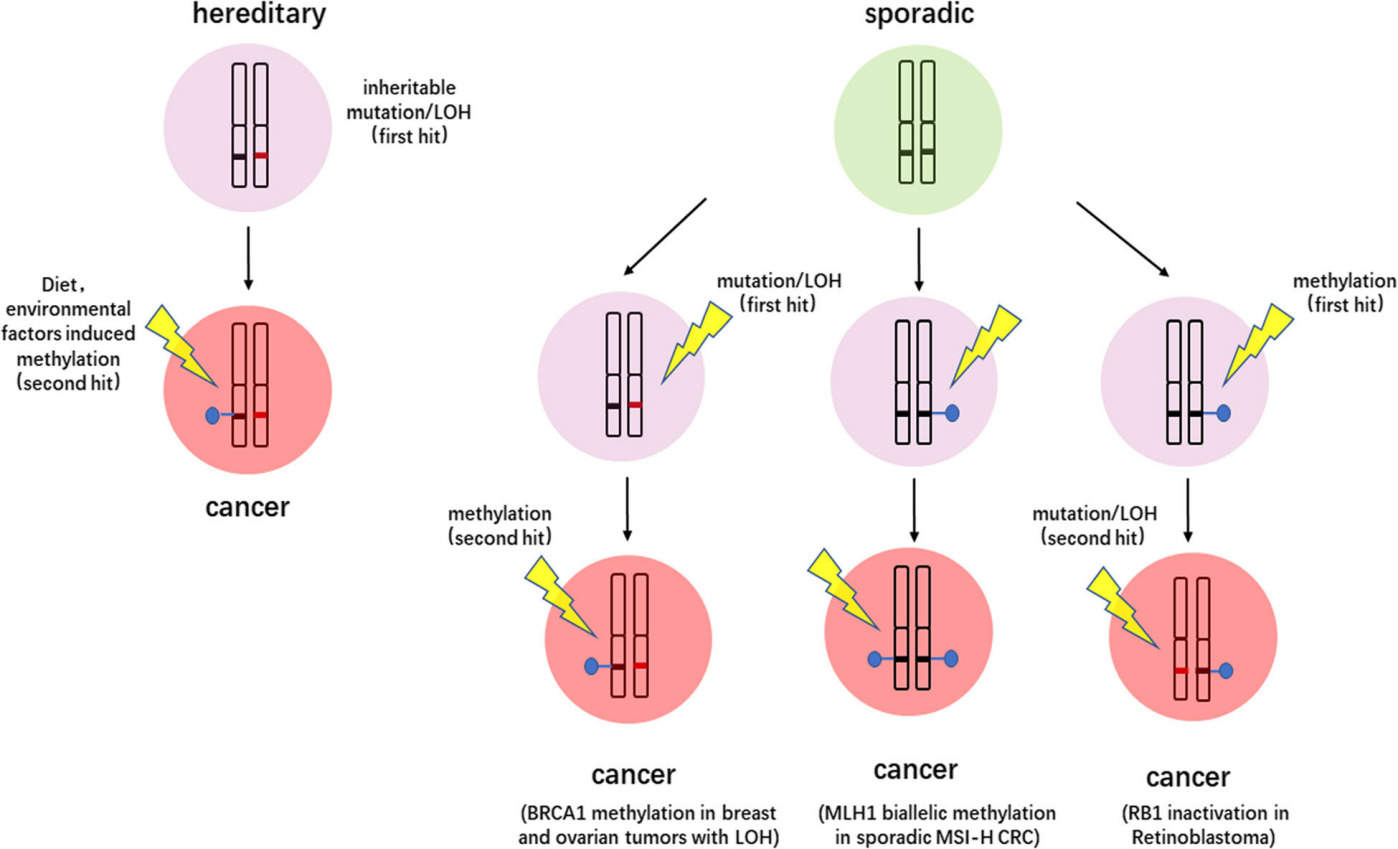
Epigenetics joins Knudson’s two hit theory. Epigenetic silencing of gene expression may happen to one allele or two alleles to join Knudson’s two hit theory. LOH, Loss of heterozygosity; , mutation or LOH; , DNA methylation

**Fig. 2:**
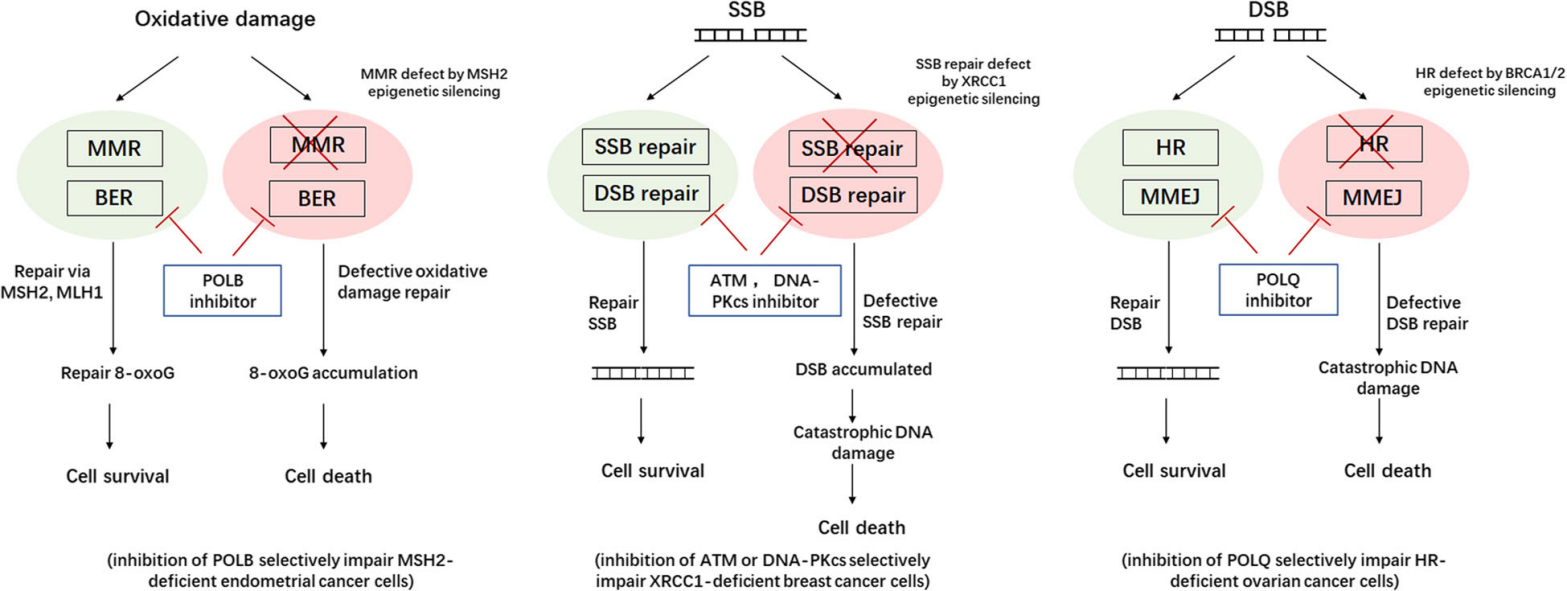
Synthetic lethality based on epigenetic defects. A. Synthetic lethality induced by combined BER inhibitor and epigenetic inactivation of MMR (inhibition of POLB selectively impair MSH2-deficient endometrial cancer cells). B. Synthetic lethality induced by combined DSB repair inhibitor and epigenetic inactivation of SSB repair (inhibition of ATM or DNA-PKcs selectively impair XRCC1-deficient breast cancer cells). C. Synthetic lethality induced by combined MMEJ repair inhibitor and epigenetic inactivation of HR repair (inhibition of POLQ selectively impair HR-deficient ovarian cancer cells). BER, base excision repair; MMR, mismatch repair; POLB, polymerase theta; SSBs, single-strand breaks; DSBs, double-strand breaks; HR, homologous recombination repair; MMEJ, microhomology mediated end joining; POLQ, polymerase theta

Based upon observations on 48 cases of retinoblastoma and published reports, Knudson found that biallelic inactivation of the gatekeeper tumor suppressor gene is necessary for initiation of tumorigenesis. This finding is called ‘Knudson’s two hit theory’, and this theory has served as an illuminating paradigm to guide the investigations of the countless tumors. In the other word, initiation of carcinogenesis needs ‘loss-of-function’ in both alleles of tumor suppressor [171, 172]. It is similar to gene mutations that epigenetic silencing of tumor suppressor genes may cause inactivation or ‘loss-of-function’ in these genes. Thus, epigenetics joins Knudson’s two hit theory (Fig. 1). DDR and cell cycle regulator genes were found frequently methylated in human cancers. It is reasonable to apply the aberrant epigenetic changes to synthetic lethal therapy in human cancers (Fig. 2).

## Combined epigenetic and genetic disruption of gene expression in DDR for synthetic lethal therapy

Mutational inactivation of gene expression or epigenetic silencing of gene expression may happen to two alleles by the same manner or by two different ways in each allele. For synthetic lethal therapeutic study, both genetic and epigenetic factors need to be included. Inactivation of MMR results in MSI. In population-based studies, the prevalence of MSI among CRCs is approximately 15%. Germline MMR mutations that give rise to hereditary non-polyposis colorectal cancer (HPNCC) account for ~ 3% of CRCs. In contrast to HPNCC, sporadic cancers are rarely found to have mutations in the MLH1 or MSH2 genes. Promoter region methylation accounts for 80-90% of MLH1 biallelic inactivation in sporadic MSI-H CRC [6, 173]. Heterozygous germline mutations in BRCA1/2 are responsible for a large fraction of hereditary breast cancers. While BRCA1/2 mutations affect a minority of breast cancer patients (fewer than 5%). BRCA1 and BRCA2 were silenced by promoter methylation in 9 and 2% of sporadic breast cancer respectively [6, 174]. Loss of heterozygosity (LOH) also joins the biallelic inactivation. For example, BRCA1 locus is lost by LOH in one allele and methylation is happened to BRCA1 in another allele [175, 176]. MGMT is mutated in 17.5% and methylated in 44% of human esophageal squamous cell carcinoma [177, 178]. Mutation of MGMT is very rare in CRC, while the methylation rate is 40% [179, 180]. WRN is frequently methylated in various cancers, while is rarely mutated [118, 181]. DNA methylation may be served as ‘second hit’ for carcinogenesis in hereditary cancer, or served as ‘first hit’ or both ‘first and second hit’ in sporadic cancer. Combination of aberrant genetic and epigenetic changes of DDR may be more efficient for synthetic lethal therapy.

## Conclusion and future perspective

The successful development of PARPi for BRCA mutant cancers provides proof-of-concept that synthetic lethality interactions can be translated into cancer therapies. A number of lessons can be learned from the discovery and the development of the PARPi in BRCA defect synthetic lethality. Such as, PARPi resistance has been widely reported in clinic, and certain percentage of patients with wild-type BRCA can still benefit from PARPi treatment. This may be explained by unknown of methylation status of BRCAs and defects of other DDR and related genes. The major issue of synthetic lethality therapy is to find good biomarkers and these markers can be used to stratify patients. Ideally, the design and interpretation of clinical trials based on synthetic lethality interactions should be based on the biological hypothesis and robust preclinical data. Therapeutic successes obtained with synthetic lethality demonstrate that DNA repair can be considered as a therapeutic target. Moreover, the concept of synthetic lethality could be extended to interactions between DNA repair deficiencies and other cell signaling abnormalities, such as aberrant genetic/epigenetic changes induced loss-of-function in tumor suppressors. Cell culture based screening approaches, patient-derived xenografts and genetically engineered mouse models of cancer will probably remain essential for uncovering synthetic lethal interactions between genomic/epigenomic lesions and selective inhibition of individual cell cycle regulators. Novel treatment modalities that can target multiple components of the same pathway may help to achieve a more sustained therapeutic benefit.

DDR pathways are the fundamental basis for maintenance of normal cells and, unfortunately, not unique to cancer. Thus, the challenge facing the efficiency of DDR inhibitors is the issue of predictive biomarkers. Some genomic/epigenomic alterations of DDR molecules are known in cancers, and some of them have been proven to be effective and specific targets for certain cancer types. Platinum-based agents could be used as mediators leading the cell to a synthetic lethality favorable for DDR-targeting agents. In addition to PARP inhibitors, new inhibitors that interfere with the activities of important genes and enzymes of the DNA damage repair pathways could also be used for the same purposes. As evolving drug resistance is inevitable until complete tumor ablation is achieved, combined targeting therapies are required. Combination of PARP inhibitors and other DDR inhibitors also have great potential synthetic lethality efficiency. Synthetic lethality only occurs when the primary pathway is defective and the backup rescue pathway is repressed. It is also important to identify synthetic rescue pathways and develop strategies to block these before therapeutic resistance develops. It is clear that while drugging DNA repair is still in its infancy, there is enormous potential to this approach because it will be applicable to many other malignancies besides those with BRCA1/2 mutations. One of the biggest problems of synthetic lethality is how to identify biallele inactivated DDR markers in human tissue samples.

As epigenetic abnormalities are apparent in the early stage of carcinogenesis and premalignant status, we may be able to develop strategies for cancer prevention. While the major issue of cancer epigenomes is technique limitation. Genetic/epigenetic heterogeneity is another major issue of targeting therapy. Intratumor heterogeneity may explain the difficulties encountered in the validation of oncology biomarkers and prediction of therapeutic resistance owing to sampling bias. Ideally, we could always target druggable trunk mutations/aberrant epigenetic changes, and then add drugs to target emerging subclones. The tumor environment may represent as much as 90% of some tumor samples. Epigenetic modifications are dynamic and responsive to environmental pressures, and they may reflect the potential of the tumor to respond to an environmental or therapeutic pressure. With the development of new techniques, combined detection of both genetic and epigenetic markers will hopefully improve the efficiencies of targeting therapy in human cancer.

## Data Availability

The material supporting the conclusion of this review has been included within the article.

## Abbreviations

SSBs: Single-strand breaks
DSBs: Double-strand breaks
ROS: Reactive oxygen species
DDR: DNA damage repair/response
BER: Base excision repair
NER: Nucleotide excision repair
HR: Homologous recombination repair
NHEJ: Non-homologous end joining repair
MMR: Mismatch repair
MMEJ: Microhomology mediated end joining
ssDNA: Single-stranded DNA
RPA: Replication protein A
siRNA: Small interfering RNA
ATM: Ataxia Telangiectasia Mutated
ATR: Ataxia Telangiectasia and Rad3 related
Chk1: Checkpoint kinase 1
Chk2: Checkpoint kinase2
PARPi: PARP inhibitors
β-NAD +: β-nicotinamide adenine dinucleotide.
RECIST: Response Evaluation Criteria in Solid Tumors
DAISY: Data mining synthetic lethality identification pipeline
SDL: Synthetic dosage lethal
WEE1i: Wee1 inhibitor
IPMN: Intraductal papillary mucinous neoplasms
CRC: Colorectal cancer
NSCLC: Non-small cell lung cancer
XRCC1: X-ray repair cross-complementing gene 1
AML: Acute myeloid leukemia
MSI: Microsatellite instability
HPNCC: Hereditary non-polyposis colorectal cancer
HCC: Hepatocellular carcinoma
ESCC: Esophageal squamous cell carcinoma
LOH: Loss of heterozygosity
POLB: Polymerase theta
POLQ: Polymerase theta
